# Description and Demo of the Best Practice Caregiving Database on 44 Research Proven Dementia Caregiving Programs

**DOI:** 10.1093/geroni/igab046.025

**Published:** 2021-12-17

**Authors:** David Bass, Alyssa Ciancibello, Rachel Schaffer, Sara Powers

**Affiliations:** Benjamin Rose Institute on Aging, Cleveland, Ohio, United States

## Abstract

A major advance in family caregiving has been the development, testing, and community delivery of research-proven, evidence-based support programs for family or friend caregivers of persons living with dementia. This presentation showcases and demos Best Practice Caregiving (BPC), a new online resource with comprehensive profiles for 44 of the top evidence-based dementia caregiving programs that are ready for scaling in communities. For these 44 programs, BPC is a database that presents key research findings with links to all its published articles, comprehensive program descriptions including all implementation features, and survey data on program delivery experiences from 324 healthcare and community organizations that offered the program as a regular part of their service portfolio 2019. BPC enables professionals to make side-by-side comparisons of the 44 programs, with the goal of increasing implementations of these evidence-based programs by healthcare and community service organizations.

